# Nicotinamide Mononucleotide Adenylyltransferase 2 (*Nmnat2*) Regulates Axon Integrity in the Mouse Embryo

**DOI:** 10.1371/journal.pone.0047869

**Published:** 2012-10-17

**Authors:** Amy N. Hicks, Diego Lorenzetti, Jonathan Gilley, Baisong Lu, Karl-Erik Andersson, Carol Miligan, Paul A. Overbeek, Ronald Oppenheim, Colin E. Bishop

**Affiliations:** 1 Wake Forest Institute for Regenerative Medicine, Wake Forest University, Winston Salem, North Carolina, United States of America; 2 Signalling Programme, The Babraham Institute, Cambridge, United Kingdom; 3 Department of Neurobiology and Anatomy and the Neuroscience Program, Wake Forest University, Winston Salem, North Carolina, United States of America; 4 The Amyotrophic Lateral Sclerosis Center, Wake Forest University, Winston Salem, North Carolina, United States of America; 5 Department of Molecular and Cellular Biology, Baylor College of Medicine, Houston, Texas, United States of America; University of Iowa, United States of America

## Abstract

Using transposon-mediated gene-trap mutagenesis, we have generated a novel mouse mutant termed *Blad* (*Bloated Bladder*). Homozygous mutant mice die perinatally showing a greatly distended bladder, underdeveloped diaphragm and a reduction in total skeletal muscle mass. Wild type and heterozygote mice appear normal. Using PCR, we identified a transposon insertion site in the first intron of *Nmnat2* (Nicotinamide mononucleotide adenyltransferase 2). *Nmnat2* is expressed predominantly in the brain and nervous system and has been linked to the survival of axons. Expression of this gene is undetectable in *Nmnat2^blad/blad^* mutants. Examination of the brains of E18.5 *Nmnat2^blad/blad^* mutant embryos did not reveal any obvious morphological changes. In contrast, E18.5 *Nmnat2^blad/blad^* homozygotes showed an approximate 60% reduction of spinal motoneurons in the lumbar region and a more than 80% reduction in the sensory neurons of the dorsal root ganglion (DRG). In addition, facial motoneuron numbers were severely reduced, and there was virtually a complete absence of axons in the hind limb. Our observations suggest that during embryogenesis, *Nmnat2* plays an important role in axonal growth or maintenance. It appears that in the absence of *Nmnat2*, major target organs and tissues (e.g., muscle) are not functionally innervated resulting in perinatal lethality. In addition, neither *Nmnat1* nor *3* can compensate for the loss of *Nmnat2*. Whilst there have been recent suggestions that *Nmnat2* may be an endogenous modulator of axon integrity, this work represents the first *in vivo* study demonstrating that *Nmnat2* is involved in axon development or survival in a mammal.

## Introduction

Genes required for axonal development and neuronal survival may provide targets for the treatment of neurodegenerative disorders and for the re-innervation of tissues after injury. They may also be used to promote innervation of tissues and organs created using tissue engineering techniques. Recently, the *Nmnat* genes (*Nmnat1*, *2*, *and 3*) have been studied as potential targets based on their ability to delay Wallerian degeneration after axonal nerve damage [1]. All the *Nmnat* family members can catalyze the synthesis of NAD^+^ both in the *de novo* pathway and in the recycling pathway [2]. *Nmnat1* is ubiquitously expressed and localizes to the nucleus. *Nmnat3* shows a more restricted expression pattern and localizes to mitochondria [3]. *Nmnat2* is expressed predominantly in neurons [3], [4], [5], [6], [7]. Its protein product has been shown to localize to the trans-Golgi complex [3], [4] where it is packaged and transported down axons to the synapse [8]. In addition to differences in tissue expression and intracellular localization, there is an isoform-specific domain on each of the *Nmnat* genes [9]. In *Nmnat2* this region is palmitoylated at two cysteine residues and, when cleaved, the NAD^+^ synthesis activity of the enzyme increases significantly [4], [10]. This provides a mechanism to increase the cytosolic pool of NAD^+^ quickly in response to a stimulus like cell stress.

*Nmnat2* has been associated with axonal survival in primary sensory and sympathetic nerve cell injury models *in vitro* [7], [8]. Historically, aberrant or exogenous overexpression of the Wld^s^ protein (a fusion protein containing *Nmnat1*) or *Nmnat1* itself, has been used to protect axons from Wallerian degeneration after injury [1], [11], but more recent studies suggest that the endogenous Nmnat isoform required for the normal maintenance of healthy axons is Nmnat2 [8], [12]. This isoform can also delay Wallerian degeneration when highly overexpressed [7], [8], [13]. Studies on the *dNmnat* ortholog in Drosophila indicate that it has a protective function analogous to the mouse *Nmnat2* gene [14], [15], [16]. Recent studies show that decreased endogenous *Nmnat2* expression and function may play a role in mouse models of Alzheimer's disease and tauopathy neurodegenerative diseases [7], [17].

Using a unique mouse mutant we present *in vivo* data indicating that during embryogenesis *Nmnat2* plays an essential role in axonal growth and/or survival. In its absence, major organs and muscles are not functionally innervated resulting in perinatal lethality, which is likely due to failure of respiratory function at birth.

## Materials and Methods

### Mouse Mutant Generation and Insertion Localization

**Figure 1:**
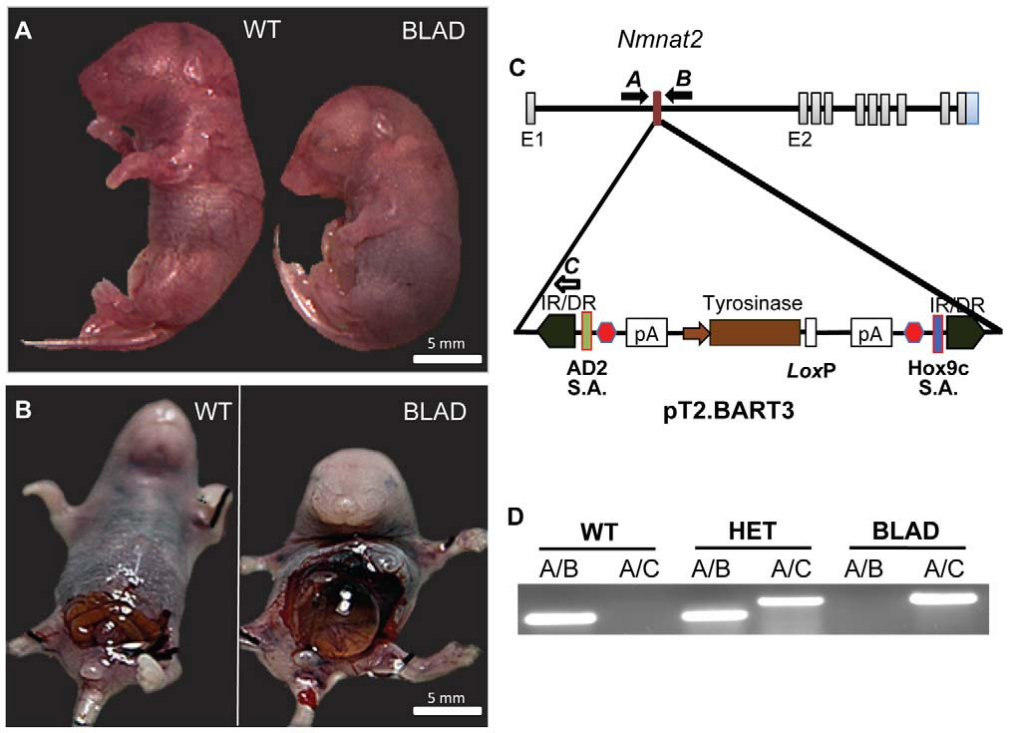
Identification of *Nmnat2* mutants and transposon localization. Images of E18.5 embryos (A, B). In both images, the wild-type (WT) is on the left and *Nmnat2^blad/blad^* (BLAD) on the right. (C) Diagram showing the localization of the pT2. BART3 transposon between exons 1 and 2 in the *Nmnat2* gene on chromosome 1. The solid arrows labeled **A** and **B** indicate the primer pair used for identification of the native gene, and the solid forward arrow **A** is combined with the hollow reverse arrow **C** to identify the presence of the transposon. IR/DR, inverted repeat/direct repeat; S.A., splice acceptor sites (Green/Blue); red hexagon, 3X stop codons; pA, synthetic Poly A signal. (D) PCR results using the primers shown in (C).

Mouse mutants were generated at Baylor College of Medicine (Houston, TX) as previously described [18]. Female transgenic mice containing a *Sleeping Beauty* transposon-based coat color gene trap construct pT2. BART3 [19] (Figure 1C) were crossed to transgenic mice carrying the SB11 transposase under the control of a 1.4-kb testis-specific human PGK2 promoter [20] After mobilization of the gene trap in the double transgenic male gametes and crossing to wild type FVB females, new coat color variants were isolated and those showing novel phenotypes were maintained as independent families. Once F1 coat color variants were identified, the transposase was bred out of the family to prevent further transposon jumping.

An asymmetric PCR amplification technique (APA) was to used to identify mouse DNA flanking transposon integration sites as previously described [19] using an APAgene kit (BioS&T Inc., Canada). Amplified bands were purified from agarose gels and directly sequenced. Sequences were compared to that of the mouse genome and PCR primers were designed to verify the presence of the *Nmnat2* insertion site. The locations of these primers are pictured in Figure 1C and their sequences are as follows; (A) Chr1F:CCATAAGAATAAATGCAGAATTAC , (B)Chr1R:TGTGCAGTTCAGTGGGTCTT , and (C)SB25:CTGGAATTTTCCAAGCTGTTTAAAGGCACAGTCAAC. Primers in the X-Y common *Kdm5d* (lysine (K)-specific demethylase 5D) gene [21] were used to sex the embryos: SmcpF: CCTGAAGCYTTTGGCTTTGAGCA and SmcpR: AAMCCRCTGCCAAATTCTTTGGA. This primer pair generates a 350bp X-specific band in males and females and a 300bp Y-specific band in males only.

### Gene Expression

Three fetuses of each genotype were obtained from timed pregnant mice at E18.5. The genotype and sex were determined using DNA extracted from the tail. The bladder, liver, kidney, lung, heart, and brain were isolated and stored in RNALater® at −20°C (Ambion cat# AM7021). The tissues were homogenized using sonication, and RNA was extracted using a QIAcube automated extraction procedure employing an RNeasy® mini plus kit (Qiagen cat# 74134). cDNA was synthesized using the Applied Biosystems High Capacity cDNA Reverse Transcription Kit (ABI cat# 4374966). TaqMan® gene expression assays for *Nmnat1, 2, and 3* (ABI assay ID# Mm01257929_m1, Mm00615393_m1, and Mm00513791_m1) were used to quantitate the levels of RNA in different tissues. The Primers PpidF1: TCGTCTTTGGACTCTTTGGAA and PpidR1: AGCGCTCACCATAGATGCTC were designed across exon junctions of the mouse *peptidylprolyl isomerase B* (*Ppib*) gene and were used as a loading/normalization control. We performed a statistical analysis on the *Pipb* qPCR data to confirm that there was no significant difference in expression across sample groups. The data are presented as the mean difference in mRNA fold expression, ± standard deviation, relative to the wild-type brain sample.

### Western Blot

Whole brains were isolated from E18.5 embryos, frozen in liquid nitrogen, and stored at −80°C. The tissue was homogenized using a sonicator on ice with 10x volume to weight of a lysis buffer containing 50 mM Tris, 150 mM NaCl, 1% Tergitol-type NP-40, 1 mM EDTA, 1 ug/mL leupeptin, 1 ug/mL aprotinin, and 1 mM PMSF. The soluble portion of the sample was quantitated using the DC™ Protein Assay (BioRad cat# 500-0116). 70 ug of total protein was run on a 10% SDS-PAGE gel before being transferred to a PVDF membrane. The membrane was probed with a mouse monoclonal antibody against NMNAT2 protein (Abcam cat# ab56980) at a 4 ug/ul concentration and β-Actin (Abcam cat# ab13822) at a 1∶1,000 dilution (control) in a 1xTBST and 5% non-fat dry milk solution with appropriate HRP tagged secondary antibodies. The SuperSignal West Pico Chemiluminescent Substrate (Thermo cat# 34080) was used to visualize the location of antibody binding which was developed on x-ray film.

### Histology and Neuron Counts

Pregnant mice were sacrificed and subjected to cesarean section at E18.5, E15.5 and E13.5. Embryos were genotyped using tail DNA. Immediately following harvest, the embryonic brains were surgically exposed and the ventral body wall was opened and eviscerated, followed by immersion in Bouin's fixative for 12–24 hours at 4°C with agitation. After processing, the head and trunk were then separately embedded in paraffin. Serial transverse or coronal sections (10–12 µm) of the thoracic through lumbar spinal cord, brain, and hind limbs were stained with either thionin or hematoxylin and eosin (H&E).

Neurons from mutant homozygous, heterozygous and wild-type (WT) embryos were counted blind in every 10^th^ section through the entire lumbar (L) spinal cord and facial nucleus (motoneurons, MNs) and in every 5^th^ section through the L4 DRG (sensory neurons). The criteria used for counting both healthy-appearing and pyknotic (apoptotic) neurons have been previously described in detail and shown to generate cell numbers comparable to unbiased stereological methods [22]. Because cell numbers in the populations of sensory and motoneurons examined here did not differ statistically between WT *vs*. heterozygote mutant mice, these data were combined for comparisons with homozygote mutant mice.

### Immunohistochemistry

For immunohistochemistry, a separate group of E18.5, E15.5 and E13.5 embryos were dissected, as described above, and immersion fixed in 2% paraformaldehyde in 0.1 M phosphate-buffered saline, pH 7.4, overnight at 4°C. Following cryoprotection in 20% sucrose overnight, the thoracic through lumbar region with attached DRG and hind limb was isolated, immersed and then frozen in a solution of 20% sucrose mixed with Tissue-TEK OCT embedding medium (3∶2). Serial sections (30 µm) were obtained through the entire region containing the attached limbs and stored at −20°C until further processing. For labeling of peripheral sensory and motor axons, we employed an antibody that recognizes Neurofilament-L (Chemicon cat. #AB9568). It was used at a 1∶1000 dilution overnight at 4°C followed by an Alexaflor 488 labeled donkey anti-rabbit secondary antibody (Invitrogen cat. #A-21206) at 1∶500 dilution for 2 hours at room temperature. To assess whether the apparent loss of peripheral axons is an artifact of reduced NF expression *vs*. axon degeneration we used an independent marker of axons, the TUJ1 antibody from Covance (1∶100) that recognizes neuronal class III β-tubulin.

### Animal Dry Weight

E18.5 whole embryos were isolated and quickly frozen in pre-weighed tubes using liquid nitrogen. DNA was extracted from the tails, which were removed prior to freezing, to determine the genotype of each animal. The frozen animals were then lyophilized for at least 48 hours before being weighed.

### Ethical Treatment of Animals

This study was carried out in strict accordance with the recommendations in the Guide for the Care and Use of Laboratory Animals of the National Institutes of Health. The protocol was approved by the Wake Forest University Laboratory Animal Care and Use Committee (Permit Number: A11-037). All pregnant females were euthanized with both CO_2_ and cervical dislocation. After removal from the uterus, pups were placed in PBS on ice for at least 10 min before tissues were isolated, and all efforts were made to minimize suffering.

## Results

### Transposon-Mediated Mouse Mutagenesis

The *Blad* mutant was one of many coat color variants isolated during the Sleeping Beauty (SB) transposon based mutagenesis project at the Baylor College of Medicine (P.O. and C.E.B). The transposon construct used in this study (pT2. BART3) is shown in Figure 1C. It is a variant of the Sleeping Beauty transposon [23], [24] modified to contain a proven splice acceptor at either end to ensure efficient gene trapping when inserted into an intron of a gene [19]. We also incorporated a tyrosinase minigene into the transposon to act as a coat color marker. This rescues the albinism in albino FVB mice [25] and allows the identification of novel transposition events by simply observing changes in coat color in the offspring of bigenic males. In addition, as the expression of the tyrosinase reporter is dose dependent, homozygotes can easily be distinguished from heterozygotes by coat color intensity. This eliminates the need for PCR-based genotyping and simplifies subsequent breeding programs. We restricted the mobilization of the transposon to the male germline by expressing the hyperactive SB11 transposase under the control of the testis specific PGK2 promoter. This eliminates the possibility that coat color changes were due to non-heritable somatic mutations.

### Identification of the *Blad* Mutant

In one family (OVB2172-P9KK4B), inbreeding revealed the absence of homozygotes at weaning as determined by the absence of a darker coat color and a significant rate of newborn lethality. Isolation of full term pups from heterozygous crosses yielded two distinct body postures (Figure 1A). About 25% of the pups had a distinctive hunched body posture with an enlarged abdomen suggestive of an embryo that was paralyzed *in utero* [26]. After opening the abdomens of the normal and hunched mice (Figure 1B), it was observed that the latter contained extremely distended bladders consistent with dysfunctional micturition *in utero*. The mutant was thus termed *Blad* for Bloated Bladder. Interestingly, whereas normal, near-term embryos exhibit body movements when isolated from the uterus, the homozygous *Blad* mutant embryos were never observed to move or breath.

### Transposon Localization to *Nmnat2*

Asymmetric PCR (APA) revealed the presence of three transposon insertion sites for OVB2172-P9KK4B on chromosomes 1, 8, and 9. Only the insertion in chromosome 1 was located within a gene. The transposon insertion on chromosome 1 was located in intron 1 of the Nicotinamide mononucleotide adenyltransferase 2 (*Nmnat2*) gene. Primers designed as illustrated in Figure 1C confirmed that all *Blad* mutant mice were homozygous for this insertion site (Figure 1D). Linkage analysis of the chromosome 1 insertion in *Nmnat2* in over 1,100 mice showed 100% concordance between the *Nmnat2* insertion site and the *Blad* phenotype. In contrast, neither the chromosome 8 or 9 insertions showed any linkage with the *blad* phenotype. To assess whether the integration sites on chromosomes 8 and 9 might be causing changes in endogenous gene expression, we performed RT-PCR expression analysis on all genes within 100 Kb of these insertion sites and found no evidence for altered expression levels (data not shown). Finally, the chromosome 8 insertion site has now segregated out of our *Blad* colony with no change in phenotype.

**Figure 2:**
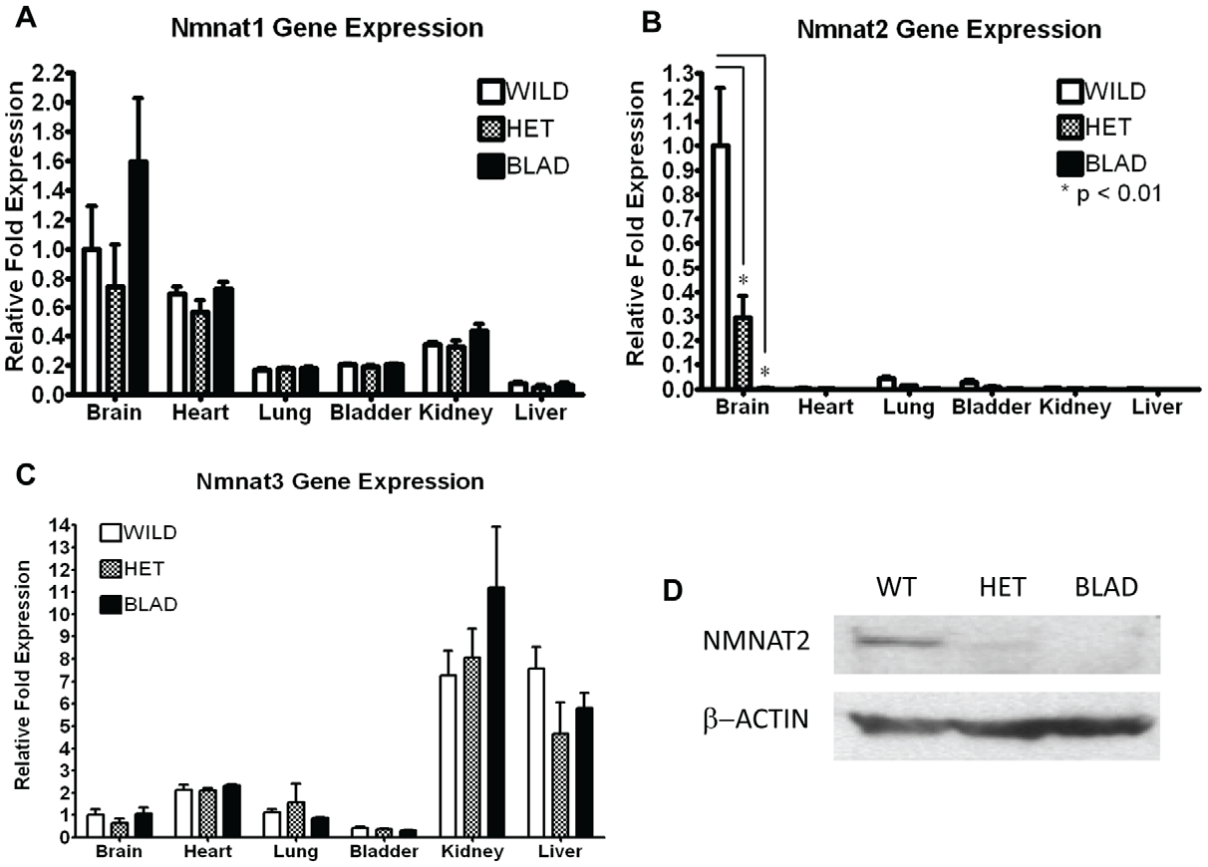
*Nmnat1*, *2*, and *3* RNA expression and *Nmnat2* protein quantification. Quantitative RT-PCR for *Nmnat1* (A), *Nmnat2* (B), and *Nmnat3* (C) using total RNA isolated from the brain, heart, lung, bladder, kidney, and liver of E18.5 embryos. Gene expression in wild-type, heterozygote (*Nmnat2^blad/+^) (HET)*, and homozygous mutant (*Nmnat2^blad/blad^*) (BLAD) mice was compared (n = 3 in each group). The data are presented as the mean difference in mRNA fold expression, ± standard deviation, relative to the wild-type brain sample. P-values were calculated using a 2-way ANOVA with a Bonferroni correction. For *Nmnat1* and *3*, there was no statistical difference in the expression levels between the 3 different genotypes observed in any of the tissues. For *Nmnat1*, all of the tissues were statistically different except brain/heart and lung/kidney. For *Nmnat3*, all of the tissues were statistically different except brain/lung and bladder/lung. (D) NMNAT2 and β-ACTIN protein levels from wild-type (WT), heterozygote (HET) (*Nmnat2^blad/+^)*, and homozygous (BLAD) (*Nmnat2^blad/blad^*) E18.5 whole brains.

To assess *Nmnat2* gene expression in different tissues, and to determine if there were differences in expression levels across the three genotypes, total RNA was isolated from 6 different tissues from three E18.5 embryos of each genotype. Quantitative RT-PCR assays confirmed that *Nmnat2* is most highly expressed in the brain (Figure 2B) as reported previously [3], [4], [5], [6], [7]. As expected, there was reduced expression of *Nmnat2* in the *Nmnat2* ^blad/+^ heterozygotes, and there was no detectable expression in the *Nmnat2* ^blad/blad^ mutant mice. NMNAT2 protein was also not detectable in the *Nmnat2* ^blad/blad^ brain (Figure 2D). There were no significant differences in *Nmnat 1* or *3* expression in any tissue from each of the three genotypes (Figure 2A, C). NMNAT2 protein levels in E18.5 brains show a pattern that is consistent with the differences seen in RNA expression across the three genotypes (Figure 2D).

### General Morphological Differences

**Figure 3:**
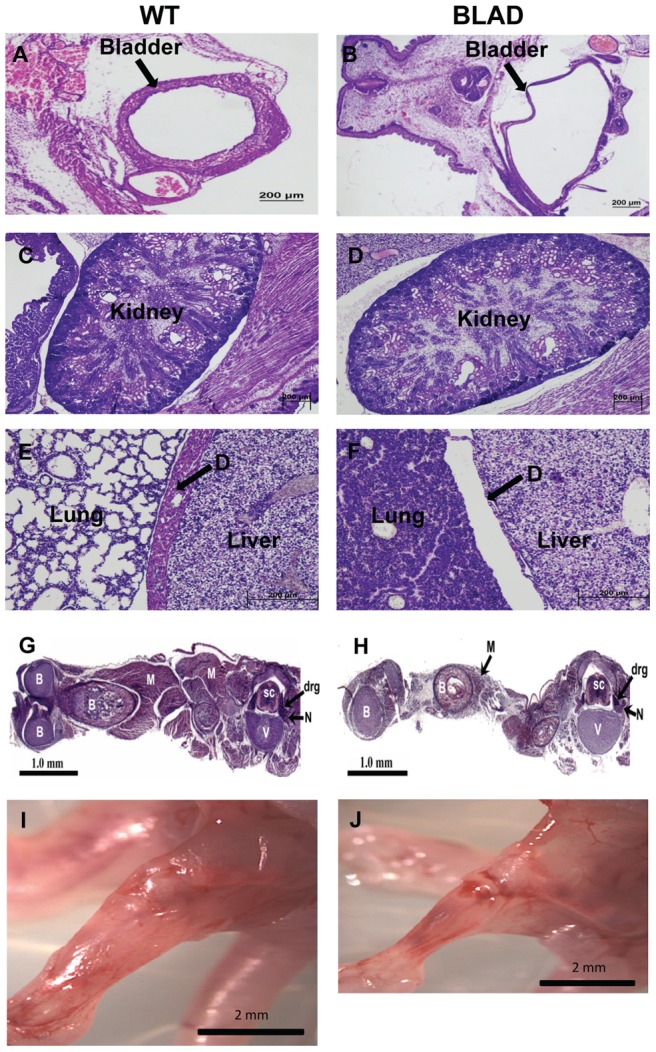
Gross morphology and histology. H & E stained E18.5 sections of wild-type (WT) (left) and *Nmnat2^blad/blad^* (BLAD) (right) embryos. (A, B) Coronal sections of the bladder. (C, D) Sagittal sections of the kidney. (E, F) Sagittal sections through the diaphragm (D). (G, H) Longitudinal sections of the hind limb and transverse sections of the lumbar region of the spinal cord. (I, J) Fresh E18.5 embryo hind limb with the skin removed. M, muscle; B, bone; N, peripheral nerve; SC, spinal cord; V, vertebra; DRG, dorsal root ganglion.

Histological sections of whole embryos were examined for gross morphological differences between the wild-type, heterozygous, and homozygous *Blad* mutant mice. Because there were no observable differences between the wild-type and heterozygotes, images of a representative wild-type mouse were used for comparison with the *Blad* mutant mice (Figure 3). We should note that for this study, our examination of the *Nmnat2* ^blad/+^ heterozygotes was limited to early developmental stages and, therefore, we cannot determine the effects of reduced *Nmnat2* expression on post natal development and aging.

The hunched body posture (Figure 1A) and distended bladder (Figure 1B, 3A, 3B) were the most obvious morphological changes in the *Nmnat2^blad/blad^* mice. At birth, the bladder was greatly distended and full of yellow liquid resembling urine, but otherwise appeared normal. Serial sections of the urogenital tract did not show any signs of a physical blockage and pressure applied to the abdomen of freshly isolated E18.5 *Blad* mutant embryos resulted in liquid exiting through the urethral opening. Analysis of the kidneys (Figures 3C, D) revealed that the *Blad* mutants had signs of underdevelopment and damage suggestive of urine reflux into the ureters and the developing kidneys. This indicated that the damage to the urogenital tract was related to urine retention, not to a physical blockage. Additionally, *Nmnat2* expression in the wild-type bladder was barely detectable (Figure 2B), but is highly expressed in the nervous tissue suggesting that the *Bloated Bladder* phenotype may be the result of innervation defects rather than structural defects in the bladder.

Since *Nmnat2^blad/blad^* mutant mice never initiate respiration, the diaphragm was another potential source of pathology. As illustrated in Figures 3E, F, the diaphragm was severely underdeveloped. Both of the mice shown in Figure 3E, F were born vaginally and had a chance to breath before being euthanized. While it is clear that the wild-type mouse was able to respire based on the open lung structure, it is equally clear that the *Nmnat2^blad/blad^* mutant mouse never respired as indicated by the collapsed lung structure. Figures 3G–J shows that in addition to the diaphragm, *Nmnat2^blad/blad^* mutants also exhibit a significant decrease in overall skeletal muscle mass consistent with the phenotype seen in other paralyzed embryos [27]. This was further reflected in the dry weight of whole E18.5 embryos: 16.54 mg +/−1.17 for wild-type and heterozygotes versus 14.54 mg +/−0.43 for the homozygous *Blad* mutants. This difference was determined to be significant with a p-value <0.009 (two tailed unpaired t-test).

Further evidence that mutation of the *Nmnat2* gene causes the observed *Blad* phenotype comes from an independently-derived *Nmnat2* gene trap mouse (obtained through EUCOMM) on a different genetic background (mixed 129/OlaHsd and C57BL/6J-*Tyr* ^c-2J^). Full characterization of this mutant is in progress, but initial observations of its gross morphology indicate that it has a phenotype that is virtually identical to the *Blad* mutant (Figure S1).

### Loss of Neurons

**Figure 4:**
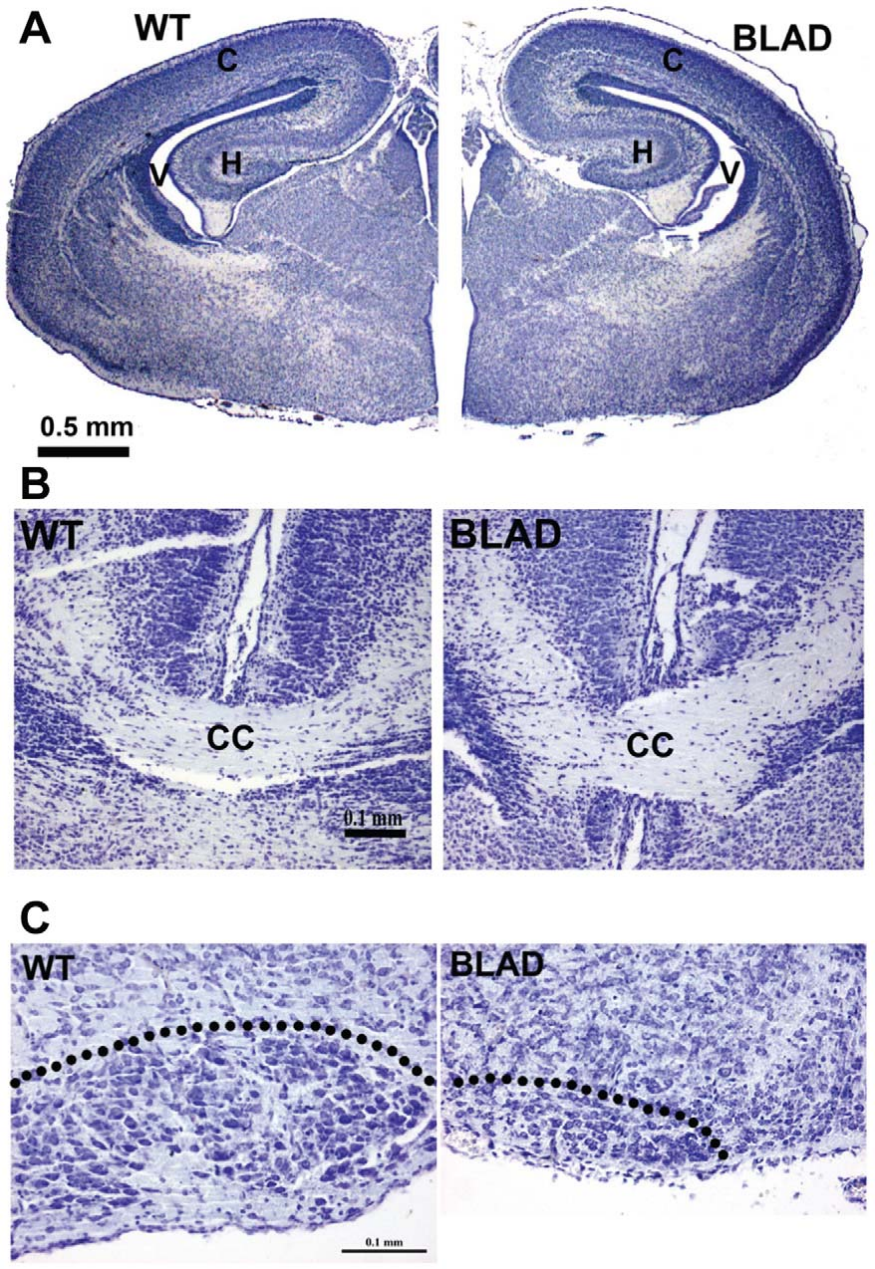
Brain and facial motor nucleus morphology. Transverse sections of E18.5 WT (left) and *Nmnat2^blad/blad^* (BLAD) (right) forebrains (A), and higher magnification views of the corpus callosum (B). Up is dorsal in (B). (C) Transverse sections of E18.5 WT (left) and BLAD (right) brainstem showing the loss of facial motoneurons (see text for quantitation). Dotted line denotes the boundary of the motor nucleus. H, hippocampus; V, ventricle; C, cortex; CC, corpus callosum.

**Figure 5:**
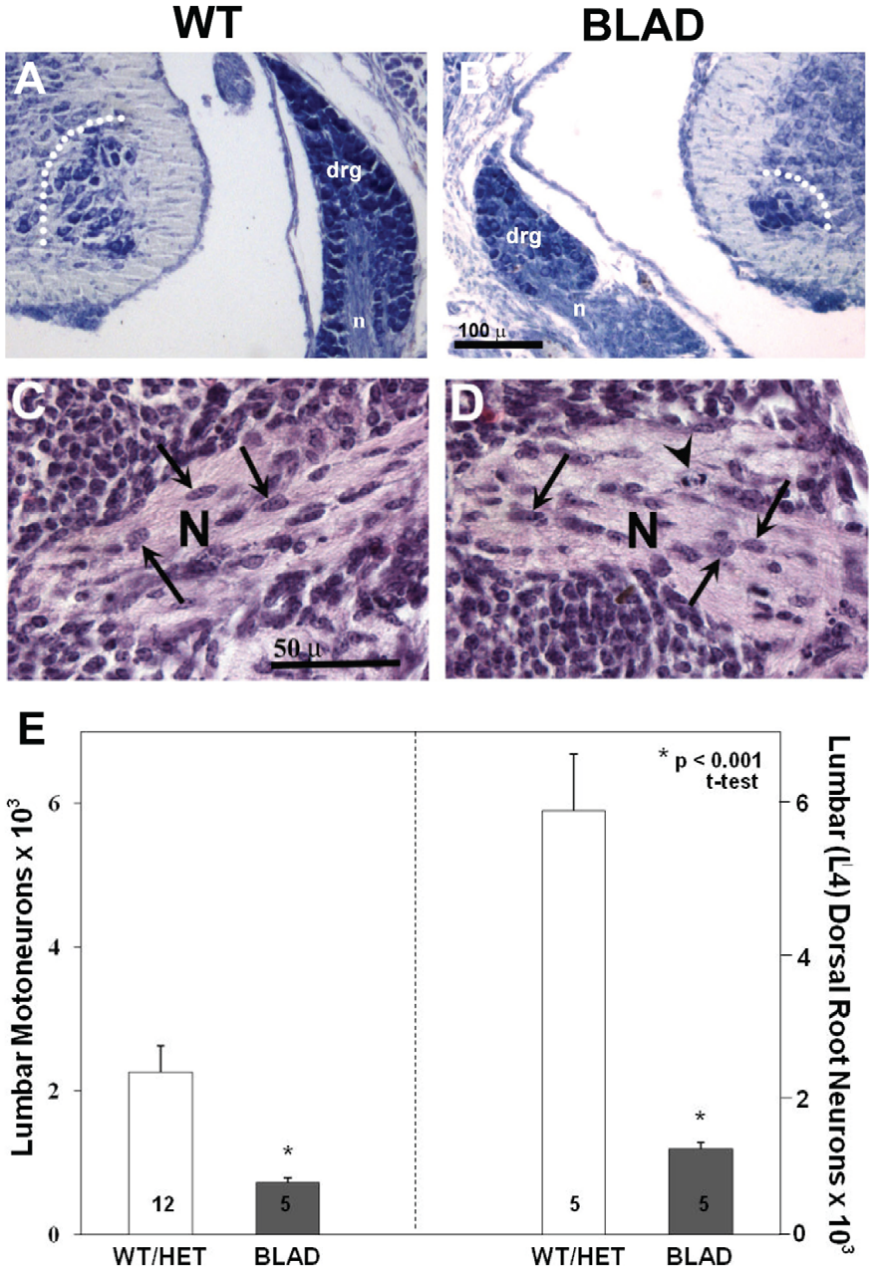
Spinal cord and dorsal root ganglion (**DRG**)**.** (A, B) Transverse sections of the lumbar spinal cord of wild-type (WT) and *Nmnat2^blad/blad^* (BLAD) mice at E18.5. Lumbar motoneurons in the spinal cord, sensory neurons in the DRG and the size of DRG are markedly reduced in BLAD mice at E18.5. (C, D) Peripheral nerves (N) from E18.5 WT (C) and BLAD (D) embryos in a lumbar region similar to that indicated by the arrows in Figures 6A–B. The lightly stained regions contain axons and the dark cells in the nerve (arrows) are presumptive Schwann cells. (E) Values (Mean ± SD) for neuron numbers for WT and *Nmnat2^blad/+^* (HET) mice did not differ and have been combined for comparison with BLAD. DRG, dorsal root ganglion; n, nerve; dotted line encloses motoneurons in the ventral horn.

Surprisingly, there were no obvious gross defects in brain morphology in *Nmnat2^blad/blad^* E18.5 embryos (Figure 4A) despite *Nmnat2* being highly expressed in this organ. Examination of the axon-rich corpus callosum also showed no obvious differences from wild-type (Figure 4B). Although the morphology of the brain stem appeared unaffected by the loss of *Nmnat2*, there was a large decrease of motoneurons in facial (Figure 4C) and other cranial motor nuclei (not shown). For example, facial motoneuron numbers at E18.5 were: WT, 3291±210 vs. BLAD, 775±39 (n = 5/group) P<0.01, t-test. Furthermore, there were no obvious changes in the overall size and shape of the spinal cord in the *Nmnat2^blad/blad^* mutants, although the DRG were greatly reduced in size (Figure 5A, B).

**Figure 6:**
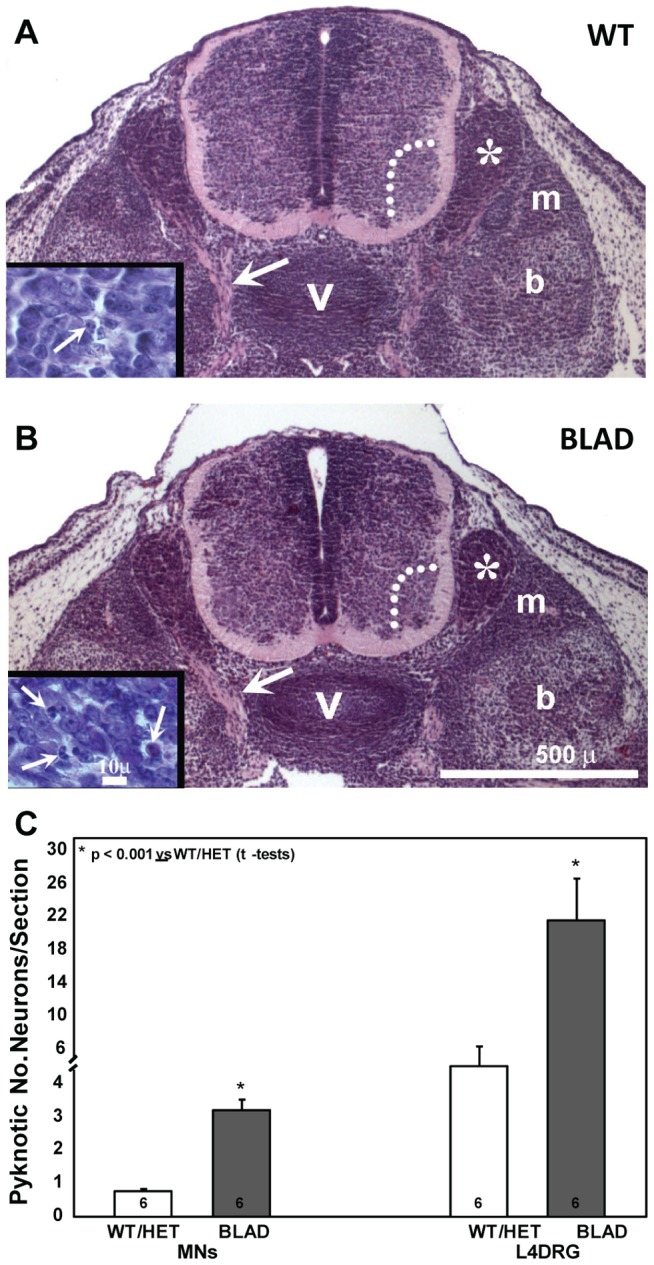
Lumbar spinal cord and pyknotic neurons at E13.5. A, B: transverse sections of caudal lumbar spinal cord of WT (A) and *Nmnat2^blad/blad^* (B) embryos. Large arrows in A and B indicate proximal axons. Insets in A and B depict pyknotic DRG cells (arrows). C: numbers of pyknotic motoneurons (MNs) and DRG sensory neurons. b, bone; m, muscle; v, vertebra; asterisk, DRG; dotted lines enclose motoneurons in the ventral horn.

In contrast, quantitation of motoneuron numbers in the spinal cord and sensory neurons in the DRG in the lumbar region on E18.5 revealed a significant reduction in both populations in *Nmnat2^blad/blad^* mutant mice (Figure 5E). Although not quantified, Schwann cells in the peripheral nerves of BLAD mutants appeared indistinguishable from WT (Figure 5C, D), making it unlikely that the loss of NMNAT2 in glia cells is involved in axon regression. Cell counts of neurons in wild-type and heterozygote mice were not statistically different, and thus were combined for this analysis. In homozygous mutants the reduction was approximately 60% for motoneurons and greater than 80% for sensory neurons in the DRG. There was a 2–3 fold increase in the number of dying (pyknotic) neurons at E13.5 in mutant embryos (Figure 6A–C) and surviving lumbar motoneuron numbers at this age were already beginning to decrease (WT, 5178±316 vs. BLAD, 4293±186 n = 5/group, p<0.05, t-test).

### Peripheral Nerve Defects

**Figure 7:**
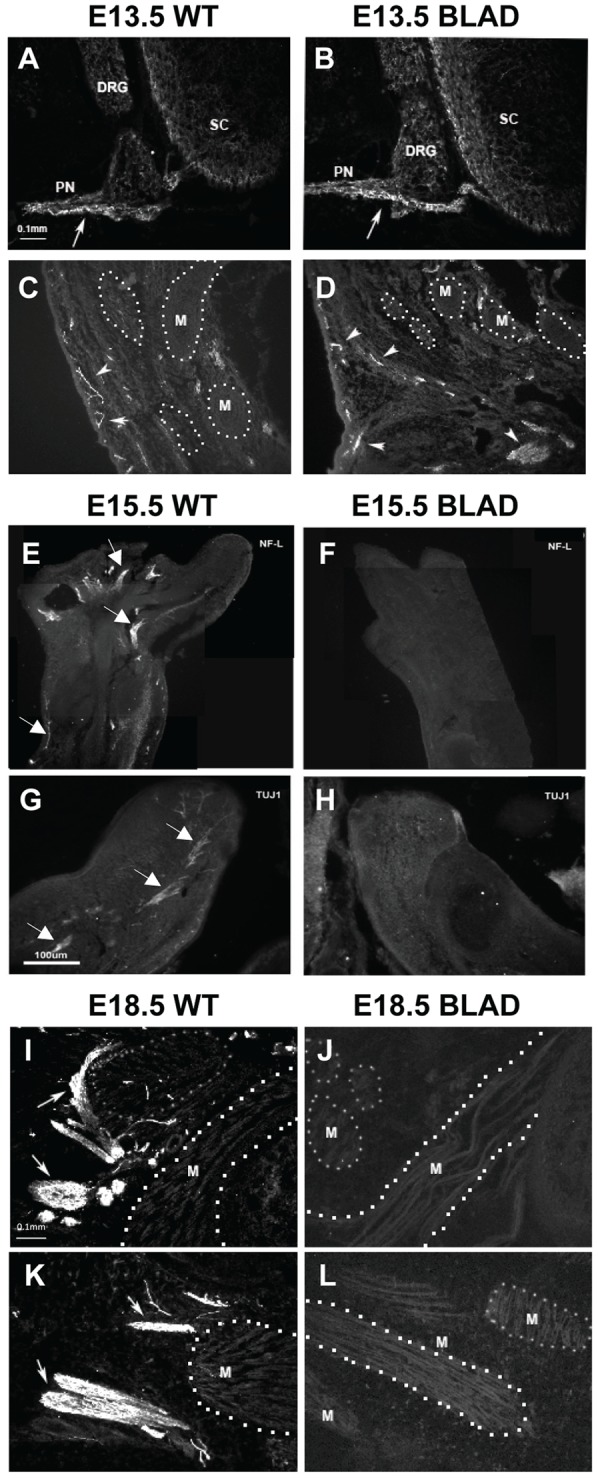
Peripheral innervation of the hind limb. Sections of spinal cord (A, B) and thigh (C, D) in E13.5 WT (A, C) and *Nmnat2^blad/blad^* (B, D) showing axons (arrows) exiting the spinal cord and innervating thigh muscles (M) in both WT and *Nmnat2^blad/blad^* mice. (Sections were stained with a neurofilament antibody and visualized by immunofluorescence). E–H, sections of the distal limb and foot in E15.5 WT (E, G) and *Nmnat2^blad/blad^* (F, H) showing NF^+^ (E, F) and TUJ1^+^ (G, H) labeled axons (arrows) in peripheral nerves. I–L, longitudinal sections of thigh muscles (M) of E18.5 WT (I, K) and *Nmnat2^blad/blad^* (J, L) mice labeled with a neurofilament antibody to visualize intramuscular axons (arrows). DRG, dorsal root ganglia; PN, peripheral nerve; SC, spinal cord; M, the unlabeled background in images of the limbs largely represents muscles many of which are bounded by a dotted line for illustrative clarity.

Spinal sensory and motor axons formed a discernible peripheral nerve close to the spinal cord in both wild-type and homozygous mutant embryos at E18.5 (Figure 5A, B). Immunostaining with a NF-L antibody revealed extensive innervation of the legs in wild-type embryos and the complete absence of NF+ axons in serial sections through the entire mutant limbs (Figure 7I–L). Because NF+ axons are present in both wild-type and mutant limbs at E13.5 (Figure 7C, D), innervation appears to be initiated but then regresses by E18.5 in *Nmnat2^blad/blad^* embryos. Even at E18.5, however, NF+ axons were present proximally in the mutant embryos (Figures 5B, 6B). Substantial NF+ axon loss in the distal leg was observed already at E15 (Figure 7E, F) which was confirmed by the use of an independent axon marker (Figure 7G, H). These observations indicate that axon loss in the leg begins between E13 and E15. Studies on whole *Nmnat2^blad/blad^* E18.5 bladders showed that the smooth muscle was functional and contained the appropriate muscarinic receptors, but there was no response to electrical field stimulation of nerves (data not shown).

## Discussion

Three transposon insertion sites were initially detected in the OVB2172-P9KK4B (*Blad*) family. Significantly, the chromosome 1 insertion into intron I of *Nmnat2* created a null mutation and was 100% linked to the *Blad* phenotype in over 1,028 offspring studied. The other two insertions on chromosomes 8 and 9 were not in genes and we were unable to find any disruption of expression in genes within 100 Kb of these insertions. Finally, the chromosome 8 insertion has segregated out of this family with no effect on phenotype. Taken together with the reported role of *Nmnat2* in axon survival *in vitro* [8] we conclude that the transposon insertion on chromosome 1 in the *Nmnat2* intron leads to a null mutation of *Nmnat2* which causes the *Blad* phenotype. This conclusion is supported by an independently-derived *Nmnat2* gene trap mouse mutant, on a different genetic background, which shows essentially the same phenotype. The combined data from these two independent gene-trap mutants indicate that loss of *Nmnat2* does indeed underlie the *Blad* phenotype.

The gross morphological analysis performed in this study suggests that the Bloated Bladder phenotype is due to lack of proper muscle innervation during development. In the absence or loss of functional innervation, the urethral sphincter appears to remain contracted and the detrusor smooth muscle remains relaxed. Thus no micturition reflex is initiated in response to bladder filling. We suggest that increased pressure in the bladder causes reflux of urine into the ureters causing damage to the kidneys which in turn may result in a developmental delay. Skeletal muscle atrophy is well known to occur in the absence of either physical or functional innervation during development [27], [28]. Defective innervation could account for the grossly underdeveloped diaphragm and the inability of homozygotes to breathe after birth. Overall, there was a significant decrease in skeletal muscle mass which was reflected in the statistically significant decreased dry weight of whole E18.5 mutant embryos.

A striking finding in E18.5 *Nmnat2^blad/blad^* mutant embryos was the marked reduction (∼60%) in spinal motoneurons and (>80%) in sensory neurons in the DRG. The reduced numbers of neurons could be due to increased programmed cell death (PCD) during the period of normal PCD between E13 and E18.5[27] or, alternatively, proliferation, specification or differentiation of neuron subtypes may be perturbed. Although further studies in progress will address this point, we favor the role of PCD, based on the increase in the number of dying (pyknotic) neurons at E13.5 in mutant embryos, and a small but significant loss of healthy appearing motoneurons at this age. The molecular basis for the extensive neuronal decrease is a critical issue for future studies.

The observation that axons in the proximal peripheral nerve, close to spinal cord, are present in mutants at E18.5, whereas in the same animals axons are absent more distally, but were present at E13.5, suggests a dying-back distal-to-proximal axonopathy. At E15.5 axons in the distal leg have disappeared whereas some proximal axons remain (not shown). These observations are consistent with the report of spontaneous Wallerian-like degeneration of the neurites of cultured sympathetic ganglia neurons that occurs in a dying-back proximal-distal pattern following knock-down of *Nmnat2* [8]. Thus, endogenous *Nmnat2* may play a critical role in the stabilization (maintenance) of peripheral axons and the prevention of axonal regression [1], [29], [30].

Embryos lacking the neuregulin receptor erbB2, that is expressed in peripheral glia, lack Schwann cells in nerves and exhibit a similar aberrant regression of peripheral axons as the *Blad* homozygotes [31]. However, we consider it unlikely that the regression of axons in our mutant embryos reflects a loss of Schwann cells. Presumptive Schwann cells [32] are present in the *Blad* homozygotes and, although the role of glia in the *Blad* phenotype has not been examined, the effects of Wld^s^ on axon survival is intrinsic to axons vs. glia. Following *in vitro* injury to neurites from Wld^s^ expressing neurons cultured in the absence of glia (Schwann cells), the transected neurites exhibit delayed Wallerian degeneration [33], [34].

The lack of obvious deficits in the gross morphology of the spinal cord and brain (CNS) in *Nmnat2* null embryos was surprising. Although we consider it to be unlikely, it is possible that *Nmnat2* is specifically only required for peripheral nerve development and maintenance [8], whereas axon survival in the CNS may be *Nmnat2* independent. In contrast to the apparent lack of CNS pathology in embryos lacking *Nmnat2*, overexpression of *Nmnat2* in the brain of larval zebrafish delayed the degeneration of injured CNS axons *in vivo* [13]. An ongoing quantitative analysis of nerve tracts and neuronal populations in mutant embryonic brains may reveal some subtle differences or, alternatively, CNS deficits may not be manifested until after birth. To address this issue, we are currently creating a brain-specific, conditional *Nmnat2* knock-out model.

The known role of all 3 *Nmnat* isoforms is NAD+ biosynthesis, indicating that *Nmnat2* mediated axon survival may require localized (e.g., axonal) NAD+ production, since unaltered expression of *Nmnat1* and *Nmnat3* does not rescue the *Blad* mutant phenotype. Currently there is no consensus on whether NAD+ production is sufficient to maintain axon integrity [35]. In fact, it has recently been shown that the selective degeneration of developing mouse DRG sensory axons requires the modulation of both an NAD+-sensitive pathway and a proapoptotic pathway, involving caspases and BAX [36]. Although NAD^+^ depletion is involved in specific types of neuronal death that require PARP-1 activation [37], we think it unlikely that the PCD we observe in the *Nmnat2* mutant embryos is dependent on this pathway [8]. Rather, motor and sensory neuronal cell death in our mutants may be indirect, and involve an apoptotic signaling pathway, following the regression of peripheral axons that results in a loss of target-derived neurotrophic support [38].

Our studies on the expression pattern of *Nmnat2* reveal a profile similar to that previously reported [3], [4], [5], [6], [7]. Expression of *Nmnat2* in the homozygous *Blad* mutant mice is undetectable. RT-PCR data show that expression in heterozygous mice is reduced by 50–75%. The fact that the *Nmnat2* protein levels in the three genotypes reflects the pattern observed in the RNA expression, suggests that this level of expression is sufficient for normal survival, at least up to the age of 4–6 months the oldest ages that we examined in this study. Interestingly, it has recently been reported that the progression of Alzheimer's disease in the APPswe/PS1dE9 transgenic mouse model and in a mouse model of tauopathy correlates with a decrease in *Nmnat2* expression [7], [17]. Cross-breeding studies can now be performed to further assess these correlations. In addition, we are beginning to investigate the consequences of reduced *Nmnat2* expression in the *Nmnat2* ^blad/+^ heterozygotes during postnatal development and aging of the CNS and peripheral nerves.

In summary, we have carried out an initial *in vivo* characterization of a neuronal phenotype in fetal mice lacking expression of *Nmnat2*. Because another family member, *Nmnat1*, is part of a fusion protein that protects axons following injury in the slow Wallerian degeneration (Wld^s^) mouse mutant, there is considerable interest in the putative functional role of *Nmnat*s in the nervous system [1]. Although many important aspects of *Nmnat2* function in the nervous system remain to be investigated [8], our observations provide the first *in vivo* evidence that endogenous *Nmnat2* is required for normal mammalian nervous system development and for the maintenance of axon integrity.

Our data suggest that neuronal growth and axon extension are initially relatively normal but then, in the absence of *Nmnat2*, axon regression leads to a loss of muscular innervation, resulting in paralysis and a fatal embryonic neuropathy.

## Supporting Information

Figure S1 **Phenotype of an** ***Nmnat2*** **gene-trap mouse derived from ES cell clone EUCE0262a08 obtained from the European Conditional Mouse Mutagenesis Program** (**EUCOMM**)**.** A, representative image of a heterozygous *Nmnat2* ^+/gt^ (+/gt) pup and a homozygous *Nmnat2* ^gt/gt^ (gt/gt) pup just after birth (P0). Homozygous *Nmnat2* ^gt/gt^ pups died at birth, due to a failure to initiate respiration (their lungs remained uninflated), and showed the same distinctive hunched posture as *Blad* mutant pups. As with the *Blad* mutant, hunched posture was already evident in E18.5 embryos which showed signs of paralysis. Heterozygous *Nmnat2* ^gt/+^ pups and embryos were indistinguishable from wild-types in this respect. B, representative bladders dissected from fixed wild-type (+/+), heterozygous *Nmnat2* ^+/gt^ (+/gt), and homozygous *Nmnat2* ^gt/gt^ (gt/gt) E18.5 embryos. *Nmnat2* ^gt/gt^ E18.5 embryos and P0 pups have massively swollen bladders – the defining feature of the *Blad* mutant. In contrast, *Nmnat2* ^+/gt^ E18.5 embryos and P0 pups have normal bladders. Finally, like the *Blad* mutant, skeletal muscle mass appeared to be reduced in homozygotes, but not in heterozygotes, at E18.5 and P0 (data not shown).
(TIF)
