# Supplementary figures and images for: Nicotinamide Mononucleotide Adenylyltransferase 2 (Nmnat2) Regulates Axon Integrity in the Mouse Embryo
Source: PLoS One. 2012 Oct 17;7(10):e47869. doi: 10.1371/journal.pone.0047869 (PMC3474723; doi:10.1371/journal.pone.0047869)

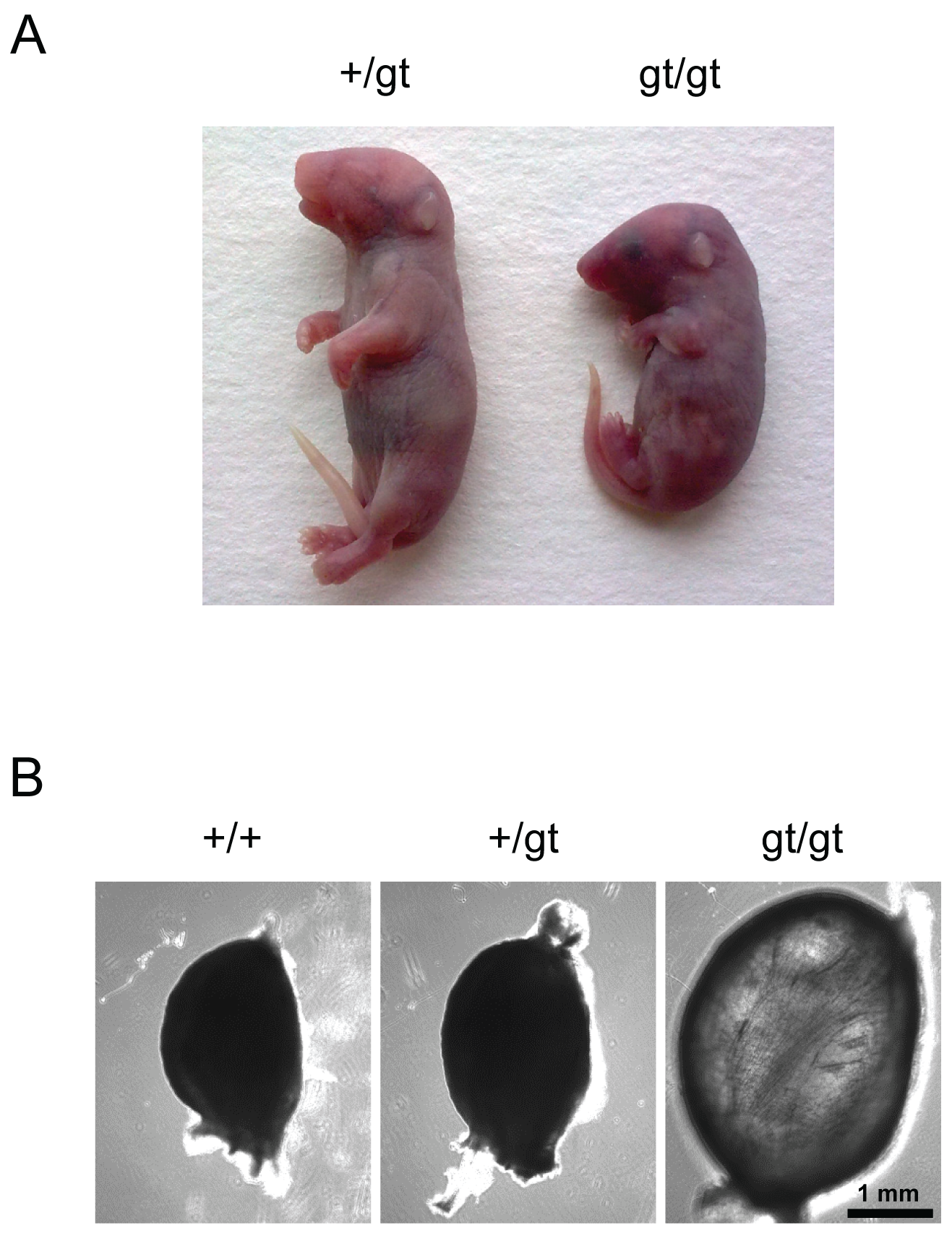

Supplement: Figure S1 — Phenotype of an Nmnat2 gene-trap mouse derived from ES cell clone EUCE0262a08 obtained from the European Conditional Mouse Mutagenesis Program (EUCOMM). A, representative image of a heterozygous Nmnat2 +/gt (+/gt) pup and a homozygous Nmnat2 gt/gt (gt/gt) pup just after birth (P0). Homozygous Nmnat2 gt/gt pups died at birth, due to a failure to initiate respiration (their lungs remained uninflated), and showed the same distinctive hunched posture as Blad mutant pups. As with the Blad mutant, hunched posture was already evident in E18.5 embryos which showed signs of paralysis. Heterozygous Nmnat2 gt/+ pups and embryos were indistinguishable from wild-types in this respect. B, representative bladders dissected from fixed wild-type (+/+), heterozygous Nmnat2 +/gt (+/gt), and homozygous Nmnat2 gt/gt (gt/gt) E18.5 embryos. Nmnat2 gt/gt E18.5 embryos and P0 pups have massively swollen bladders – the defining feature of the Blad mutant. In contrast, Nmnat2 +/gt E18.5 embryos and P0 pups have normal bladders. Finally, like the Blad mutant, skeletal muscle mass appeared to be reduced in homozygotes, but not in heterozygotes, at E18.5 and P0 (data not shown). (TIF) [file pone.0047869.s001.tif]
